# DMDtoolkit: a tool for visualizing the mutated dystrophin protein and predicting the clinical severity in DMD

**DOI:** 10.1186/s12859-017-1504-4

**Published:** 2017-02-02

**Authors:** Jiapeng Zhou, Jing Xin, Yayun Niu, Shiwen Wu

**Affiliations:** 1 Department of Neurology, General Hospital of Chinese People’s Armed Police Forces, Beijing, 100039 China; 2 Precision Medical Laboratory, General Hospital of Chinese People’s Armed Police Forces, Beijing, 100039 China

**Keywords:** Duchenne muscular dystrophy (DMD), Becker muscular dystrophy (BMD), Assisted diagnosis, Ambush hypothesis, Hidden stop codons

## Abstract

**Background:**

Dystrophinopathy is one of the most common human monogenic diseases which results in Duchenne muscular dystrophy (DMD) and Becker muscular dystrophy (BMD). Mutations in the dystrophin gene are responsible for both DMD and BMD. However, the clinical phenotypes and treatments are quite different in these two muscular dystrophies. Since early diagnosis and treatment results in better clinical outcome in DMD it is essential to establish accurate early diagnosis of DMD to allow efficient management. Previously, the reading-frame rule was used to predict DMD versus BMD. However, there are limitations using this traditional tool. Here, we report a novel molecular method to improve the accuracy of predicting clinical phenotypes in dystrophinopathy. We utilized several additional molecular genetic rules or patterns such as “ambush hypothesis”, “hidden stop codons” and “exonic splicing enhancer (ESE)” to predict the expressed clinical phenotypes as DMD versus BMD.

**Results:**

A computer software “DMDtoolkit” was developed to visualize the structure and to predict the functional changes of mutated dystrophin protein. It also assists statistical prediction for clinical phenotypes. Using the DMDtoolkit we showed that the accuracy of predicting DMD versus BMD raised about 3% in all types of dystrophin mutations when compared with previous methods. We performed statistical analyses using correlation coefficients, regression coefficients, pedigree graphs, histograms, scatter plots with trend lines, and stem and leaf plots.

**Conclusions:**

We present a novel DMDtoolkit, to improve the accuracy of clinical diagnosis for DMD/BMD. This computer program allows automatic and comprehensive identification of clinical risk and allowing them the benefit of early medication treatments. DMDtoolkit is implemented in Perl and R under the GNU license. This resource is freely available at http://github.com/zhoujp111/DMDtoolkit, and http://www.dmd-registry.com.

**Electronic supplementary material:**

The online version of this article (doi:10.1186/s12859-017-1504-4) contains supplementary material, which is available to authorized users.

## Background

Duchenne muscular dystrophy (DMD) is an X-linked recessive disorder caused by dystrophin gene mutations [1]. It occurs in boys with an incidence rate of 1/3500 [2, 3]. DMD patients usually show symptoms between 3 and 5 years old. They tend to lose ability to walk by age 12 years and succumb to cardiopulmonary failure from late teens to early 20s. Both DMD and BMD (a milder phenotype) are caused by mutations in the dystrophin gene. Dystrophin is the largest gene in human genome, spaning 2.4 Mb and containing 79 exons. The full-length transcript expressed in human skeletal muscle encodes a protein of 3685 amino acids, which gives rise to a 427 kDa dystrophin protein (Dp427m) that links cytoskeletal actin to the extracellular matrix via the sarcolemmal dystrophin-associated glycoprotein complex (DGC). Dp427m is composed of four domains: an amino-terminal actin-binding domain (ABD), a central rod domain that contains spectrin-like repeats, a cysteine-rich domain, and a unique carboxy-terminal domain.

The theory currently used to predict whether a mutation will result in a DMD or BMD phenotype is the reading-frame rule (Monaco rule): “Adjacent exons that can maintain an open reading frame (ORF) in the spliced mRNA despite a deletion event would give rise to the less severe BMD phenotype and predict the production of a lower molecular weight, semifunctional dystrophin protein. Adjacent exons that cannot maintain an ORF because of frame shifted triplet codons would give rise to the more severe DMD phenotype due to the production of a truncated, nonfunctional dystrophin protein [4]”. In-frame mutations, such as deletion of exons 45-47 whose length is 474 bp (i.e., 158 codons), would maintain the ORF and usually lead to BMD. In the case of DMD, the well-known types of DMD-causing mutations include large mutations [large deletions (larger than 1 exon), large duplications (larger than 1 exon)], small mutations [small deletions (less than 1 exon), small insertions (less than 1 exon)], splice site mutations (less than 10 bp from exon), point mutations (nonsense, missense), and mid-intronic mutations. Large deletions, such as deletion of exon 45 whose length is 176 bp (causing frameshift), are the most commonly observed and account for about 68% of the total mutations. The second common mutation is large duplications, such as duplication of exon 2, that account for about 11% [5]. Large deletions usually occur in the rod domain while large duplications mostly occur in the ABD domain.

Currently there are a number of databases reporting correlation between DMD genotype and phenotype. These include the Leiden muscular dystrophy pages (http://www.dmd.nl/) in the Netherlands [6], the UMD-DMD (http://www.umd.be/DMD/) [7], the eDystrophin (http://edystrophin.genouest.org/) in France, and the TREAT-NMD DMD Global database (http://umd.be/TREAT_DMD/) in Belgium [5]. They offer a web-based query for existing mutations, showing their effects on the function of dystrophin gene and protein, and the frequency of each mutation. Although eDystrophin correlates information between protein isoforms and structures with pathology phenotypes it only shows structure of dystrophin protein and phenotype distribution for existing in-frame mutations. The small insertions or deletions to the splice sites of dystrophin gene appear to follow the reading-frame rule, but it is sometimes difficult to apply to a novel mutation or a nonsense mutation or a combination of multiple mutations. Furthermore, exceptions to the reading-frame rule have been widely reported. Given this limitation, we considered the potential underlying mechanisms of DMD and proposed using several other rules or patterns such as “ambush hypothesis” [8], “hidden stop codons” [9] and “exonic splicing enhancer (ESE)” [10, 11] to distinguish between DMD and BMD of various types of mutations.

We previously built a Registration Network of Genetic Diseases database in China (www.dmd-registry.com) with information of more than 1400 Chinese DMD/BMD patients. We have now established a collaboration with the Lilac Garden (www.dxy.cn) according to the upcoming “One City, One Doctor Project” [12]. Lilac Garden is the leading online network and service provider in China. Our doctors and researchers in the field of clinical medicine and life sciences are establishing close working relationships with patients to improve the established database. These are important for developing an effective management team in the field. Early and accurate diagnosis is key to an effective treatment.

In the present study, we developed a computer software DMDtoolkit, which was based on Perl (Practical extraction and reporting language) and R environment, to provide an aid to the diagnosis of DMD. We also took into the consideration of other molecular characteristics such as mutated protein structure, pedigree of DMD family, and frequency of mutations. The DMDtoolkit is provided in the Additional files and can be downloaded from http://github.com/zhoujp111/DMDtoolkit or http://www.dmd-registry.com after registration.

## Implementation

The DMDtoolkit (including DMDtoolkit.pl, DMDtoolkit.R, etc.) was designed to Perl and R by the Department of Neurology in the General Hospital of Chinese People’s Armed Police Forces. It is a free software for statistical computing and graphics. DMDtoolkit is a tool for analyzing the dystrophin mutations, predicting structure and features of the disordered protein, and visualizing statistical and genetic test results. It can help the clinicians and patients to better understand DMD.

### Environment

Perl is a scripting language first created by Larry Wall to be used as a supplement to the programming which is freely available for download and general use [13]. R is a language and environment for statistical computing and graphics, also freely available for download and academic use [14]. These two platforms can be used jointly to quickly and effectively analyze and visualize the data. All codes were designed using ActivePerl version 5.16.2 and R version 3.0.2 on Windows 10 Professional platform. We refer the reader to the section ‘Availability and Requirements’ at the end of this article, which is a summary of the software involved in this version of DMDtoolkit.

### Data analysis and visualization framework

#### Smartly screening of incomplete data

We found patients’ medical records are often incomplete in the clinical indicators for DMD patients. In order to maximize the use of existing data, a module of automatic screening was developed. An example is provided in Table 1.

**Table 1:** An example of incomplete data

ID	Visit	Age	BMI	LVEDD	SNIP	9 m	WISC
1	1	8.5	19.5	37		11.3	
1	2	9.2		41	51	20.5	
1	3	9.7	25.8		50		85

This table shows an example of incomplete data of a DMD boy with three visits. *BMI* body mass index, *LVEDD* left ventricular end-diastolic dimension, *SNIP* sniff nasal inspiratory pressure, *WISC* Wechsler Intelligence Scale for Children

This DMD child made three visits to the clinic and four sets of indicators were collected: body mass index (BMI), left ventricular end-diastolic dimension (LVEDD), sniff nasal inspiratory pressure (SNIP) and Wechsler Intelligence Scale for Children (WISC). Due to the non-linear changes of some indicators, the imputation method might not be suitable to use [15]. However, the module named SmartScreen.R was coded with imputation method based on random forest which was a type of ensemble machine learning algorithm. In our work, the most informative data could be to select according to weighted score which is the sum of weight value of all indicators [16]. The weight for each indicator equals to one by default and can be changed by parameter settings. One or more than one indicator of interest can be set indispensable, which means that if any of them was missing, the entire data would be discarded. We used the following formula to calculate the scores:

$$ \mathrm{Score}={\displaystyle \sum_{\mathrm{i}}^{\mathrm{j}}{\mathrm{weight}}_{\mathrm{i}}\times {\mathrm{i}\mathrm{ndicator}}_i} $$

i is the column number of the first indicator, and j is the column number of the last indicator; weight vector can be set via command or be changed in the program.

#### Assisted diagnosis for DMD/BMD

Reading-frame rule has traditionally been used to distinguish between DMD and BMD, which has been shown to hold true for about 90% of patients [5, 6]. Another two methods were later developed: the length of mutated protein [8] and the number of potential stop-gains [9]. The length of mutated protein method was initiated by ambush hypothesis. Fanin et al. emphasized the threshold effect and estimated that the size of a molecule needed to ensure the integrity and function of the dystrophin-associated glycoprotein (DAG) complex should be at least 200 kDa (about 43 exons or 2000 aa) [8]. In this study, the threshold was identified as 3000 aa, which is explained in the following paragraphs. Seligmann et al. revealed that hidden stop codons prevent off-frame gene reading, which was named potential stop-gains in our research. Thus, the number of potential stop-gains was associated with harmfulness of the mutation [9]. At this work, the transcript carrying a mutation or multiple mutations was translated into the mutated protein, and the length of mutated protein and the number of potential stop-gains were calculated. The cutoff value for the length of mutated protein was identified in DMD as less than 3000 aa or more than 3685 aa (outside of the length of normal protein). The cutoff value for the number of potential stop-gains to be regarded in DMD was ≠1, since normal protein has only one stop codon. We combined all three rules (the reading frame rule, the protein length, and the potential stop gains) to predict a DMD versus BMD. Some other rules or patterns were also applied. For example, large in-frame deletions in the central rod domain removing more than 35 exons usually led to DMD [8], and mutations in the cysteine-rich domain usually resulted in DMD [17, 18]. The effect of exonic splicing enhancer (ESE) was also considered. A file named “ESE matrices.txt” (in the Additional file 1 “codes_DMDtoolkit”) which contains the matrices of serine/arginine-rich (SR) proteins was used to predict the ESE effect. Cartegni et al. revealed that point mutations responsible for genetic diseases may cause aberrant splicing. Such mutations can disrupt splicing by directly inactivating or creating a splice site, by activating a cryptic splice site or by interfering with splicing regulatory elements [19]. A patient would be diagnosed as “DMD” by the joint predication if any method predicted him as “DMD”. False positive rate (FPR) and false negative rate (FNR) were calculated for each method. Here a “false positive” means that a “BMD” is falsely predicted as a “DMD”, while a “false negative” means that a “DMD” is falsely predicted as a “BMD”.

We made several assumptions during the data analysis on DMD/BMD prediction:

- For exon splice sites, we assumed that only the nearby exons would be skipped.
- For missing the promoter region, we assumed that it could not create a transcript.
- For a combination of multiple mutations, the 5′ mutation would be firstly considered. If the transcript was predicted to stop translating before another mutation (3′ mutation), the 3′mutation would be ignored.
- For some large deletions, several supplementary rules or patterns [8, 20, 21] for the reading-frame rule were applied.

The patient data used for this test were selected from the TREAT-NMD DMD Global database [5], Flanigan’s DMD patients [19] and DMD patients of GHCPAPF (General Hospital of Chinese People’s Armed Police Forces). The data were prepared in plain text format, such as “Flanigan’s_DMD_patients.txt” in the Additional file 2 “data_DMDtoolkit”. This research was approved by research ethics committee and medical ethics committee of General Hospital of Chinese People’s Armed Police Forces. We clearly confirmed that signed informed consents were obtained from parents of DMD/BMD children or BMD patients in adult. The mutations which cannot tell the exact change of nucleotide sequence, such as c.1335ins680, were filtered since DMDtoolkit conducts the prediction via translating nucleotide sequence to protein sequence.

#### Visualization of mutated protein

**Fig. 1:**
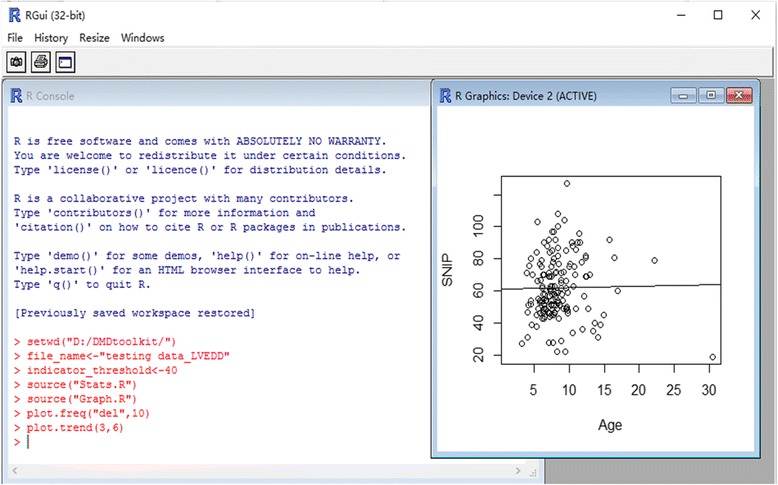
A snapshot of RGui. RGui is an interactive GUI to execute the code that displays the statistics and figures. The command plot.trend(3,6) performed in the R Console creates the figure of trend line which is displayed in the R Graphics

We drew the sequence of the mutated protein according to its mutation, motifs, and potential protein length, then applied the reading-frame rule. We also analyzed the combination of multiple mutations (such as Large duplication + Small deletion, Splice site + Nonsense). RGui was used to execute the code and display the statistics in the figures (Fig. 1). More snapshots of GUIs can be found in “codes_DMDtoolkit/Manual.docx” in the Additional file 1. Users familiar with R can also use R studio which includes a code editor, debugging and visualization tools. We selected some common mutations from TREAT-NMD database [5] as test data (totally 51 mutation types in the Additional file 2 “data_DMDtoolkit/data_diagnosis/DMDsamples.txt”): large deletions (≥1 exon) (freq ≥100), large duplications (≥1 exon) (freq ≥10), small deletions (<1 exon) (freq ≥4), small insertions (<1 exon) (freq ≥3), splice sites (<10 bp from exon) (freq ≥4), nonsense (freq ≥10). We simulated two combinations of multiple mutations with common mutations from TREAT-NMD, i.e., exon56-62dup plus c.9204_9207delCAAA and c.9563+1G>A plus c.9568C>T. In addition, there were six combinations of multiple mutations (exon5-19dup plus exon38-41dup, exon29dup plus exon45dup, exon45-55dup plus exon65-79dup, exon5-18dup plus exon19-41del plus exon42dup plus exon43-44del, exon10-16dup plus exon22-24dup, exon50-60dup plus exon63-79dup) from Flanigan’s DMD patients, and seven DMD patients from GHCPAPF (exon1del plus exon2dup plus Dp427cdel, exon31-43dup plus c.4000G>A p.Gly1334Arg, exon45del plus exon47-52del, exon50del plus exon52del, c.1898dupA plus c.5234G>A p.Arg1745His, exon1-12del plus Dp427c-490ntdel, c.7096C>A p.Gln2366Lys plus c.10101_10103delAGA p.Glu3367del). Using “c.1898dupA plus c.5234G>A p.Arg1745His” as an example, we investigated whether the frameshift c.1898dupA would change the downstream missense c.5234G>A to a nonsense. While the reading-frame rule alone was not able to answer the protein length and stop-gain number seemed to be able to avoid this problem.

#### Visualizing distribution of mutations and pedigree

Basic characteristics such as distribution of mutations in DMD patients and their pedigrees was graphed for easy understanding with its respective modules. The test data were selected from DMD patients of GHCPAPF.

### Key features and functionalities

The functions of DMDtoolkit include:

1. aided diagnosis for DMD/BMD using genetic testing
2. drawing the sequence and motifs of mutated protein
3. drawing pedigree of DMD family
4. smart screening data to maximize the use of existing data
5. performing statistical analysis for DMD population and visualizing results.

## Results

### Assisted diagnosis

DMDtoolkit was used according to four rules: reading-frame rule, length of potential protein, number of potential stop-gains, ESE rule, and several patterns on location of mutations. This created three result files: *.diag, *.diag2 and *.diag3 (in the Additional file 3 “results_DMDtoolkit/results_diagnosis/”). The differences between the three files were the extent of application of reading-frame rule and whether applying patterns or not: *.diag was restricted to exon deletions/duplications; *.diag2 was expanded to small deletions/duplications and splice sites; and *.diag3 applied ESE rule to nonsense mutations, and applied size and location info to in-frame deletions. The following results were obtained based primarily on the *.diag2 file (first four columns of statistics in Table 2) and partly on the *.diag3 file (the last column of statistics in Table 2). The detailed results can be found in the Additional file 3 “prediction results of DMD patients.xlsx” in the folder “results_DMDtoolkit/results_diagnosis/”. Based on the reading-frame rule, the accuracy, FPR (false positive rate) and FNR (false negative rate), of predicting DMD/BMD were 91.0%, NA (not applicable), 9.0% (large deletions/duplications in 5161/5681 = 90.8% for accuracy, NA for FPR, 520/5681 = 9.2% for FNR; small deletions/duplications in 475/483 = 98.3% for accuracy, NA for FPR, 8/483 = 1.7% for FNR; splice sites in 155/198 = 78.3% for accuracy, NA for FPR, 43/198 = 21.7% for FNR) in TREAT-NMD DMD patients. They were 85.0, 5.8, and 9.2% (large deletions/duplications in 369/435 = 84.8% for accuracy, 23/435 = 5.3% for FPR, 43/435 = 9.8% for FNR; small deletions/duplications in 69/78 = 88.5% for accuracy, 7/78 = 9.0% for FPR, 2/78 = 2.6% for FNR; splice sites in 17/22 = 77.3% for accuracy, 1/22 = 4.5% for FPR, 4/22 = 18.2% for FNR) in Flanigan’s DMD patients. In GHCPAPF patients they were 92.0, 0.4, and 7.6%, (large deletions/duplications in 213/231 = 92.2% for accuracy, 0/231 = 0% for FPR, 18/231 = 7.8% for FNR; small deletions/duplications in 15/17 = 88.2% for accuracy, 1/17 = 5.9% for FPR, 1/17 = 5.9% for FNR; splice sites in 1/1 = 100.0% for accuracy, 0/1 = 0% for FPR, 0/1 = 0% for FNR). According to the length of potential protein, the accuracy, FPR and FNR were 91.0%, NA, 9.0% (large deletions/duplications in 5283/5681 = 93.0% for accuracy, NA for FPR, 390/5681 = 7.0% for FNR; small deletions/duplications in 408/483 = 84.5% for accuracy, NA for FPR, 75/483 = 15.5% for FNR; splice sites in 99/198 = 50.0% for accuracy, NA for FPR, 99/198 = 50.0% for FNR) in TREAT-NMD DMD patients. They were 83.2, 6.7, 10.1% (large deletions/duplications in 374/435 = 86.0% for accuracy, 31/435 = 7.1% for FPR, 30/435 = 6.9% for FNR; small deletions/duplications in 59/78 = 75.6% for accuracy, 4/78 = 5.1% for FPR, 15/78 = 19.2% for FNR; splice sites in 12/22 = 54.5% for accuracy, 1/22 = 4.5% for FPR, 9/22 = 40.9% for FNR) in Flanigan’s DMD patients. In GAPGH group they were 92.8, 2.4, 4.8% (large deletions/duplications in 217/231 = 93.9% for accuracy, 5/231 = 2.2% for FPR, 9/231 = 3.9% for FNR; small deletions/duplications in 13/17 = 76.5% for accuracy, 1/17 = 5.9% for FPR, 3/17 = 17.6% for FNR; splice sites in 1/1 = 100.0% for accuracy, 0/1 = 0% for FPR, 0/1 = 0% for FNR). For the number of potential stop-gains, the accuracy, FPR and FNR were 91.3%, NA, 8.7% (large deletions/duplications in 5180/5681 = 91.2% for accuracy, NA for FPR, 501/5681 = 8.8% for FNR; small deletions/duplications in 476/483 = 98.6% for accuracy, NA for FPR, 7/483 = 1.4% for FNR; splice sites in 155/198 = 78.3% for accuracy, NA for FPR, 43/198 = 21.7% for FNR) in TREAT-NMD DMD patients. They were 85.2, 6.0, 8.8% (large deletions/duplications in 370/435 = 85.1% for accuracy, 24/435 = 5.5% for FPR, 41/435 = 9.4% for FNR; small deletions/duplications in 69/78 = 88.5% for accuracy, 7/78 = 9.0% for FPR, 2/78 = 2.6% for FNR; splice sites in 17/22 = 77.3% for accuracy, 1/22 = 4.5% for FPR, 4/22 = 18.2% for FNR) in Flanigan’s DMD patients. In the GHCPAPF group they were 93.6, 0.4, 6.0% (large deletions/duplications in 217/231 = 93.9% for accuracy, 0/231 = 0% for FPR, 14/231 = 6.1% for FNR; small deletions/duplications in 15/17 = 88.2% for accuracy, 1/17 = 5.9% for FPR, 1/17 = 5.9% for FNR; splice sites in 1/1 = 100.0% for accuracy, 0/1 = 0% for FPR, 0/1 = 0% for FNR). If we use 2000 aa [8] as the threshold of the length of potential mutated protein, the accuracy, FPR and FNR were 31.3%, NA, 68.7% (large deletions/duplications in 1634/5681 = 28.8% for accuracy, NA for FPR, 4047/5681 = 71.2% for FNR; small deletions/duplications in 293/483 = 60.7% for accuracy, NA for FPR, 190/483 = 39.3% for FNR; splice sites in 155/198 = 78.3% for accuracy, NA for FPR, 43/198 = 21.7% for FNR) in TREAT-NMD DMD patients. They were 44.9, 5.0, 50.1% (large deletions/duplications in 185/435 = 42.5% for accuracy, 23/435 = 5.3% for FPR, 227/435 = 52.2% for FNR; small deletions/duplications in 49/78 = 62.8% for accuracy, 4/78 = 5.1% for FPR, 25/78 = 32.1% for FNR; splice sites in 6/22 = 27.3% for accuracy, 0/22 = 0% for FPR, 16/22 = 72.7% for FNR) in Flanigan’s DMD patients. In GHCPAPF group they were 38.4, 1.9, 59.7% (large deletions/duplications in 81/231 = 35.1% for accuracy, 4/231 = 1.7% for FPR, 146/231 = 63.2% for FNR; small deletions/duplications in 10/17 = 58.8% for accuracy, 1/17 = 5.9% for FPR, 6/17 = 35.3% for FNR; splice sites in 1/1 = 100.0% for accuracy, 0/1 = 0% for FPR, 0/1 = 0% for FNR). Thus, we chose the 3000 aa as the threshold. The two new methods have a similar accuracy to the reading-frame method.

**Table 2:** Accuracy, FPR and FNR of prediction between DMD and BMD in DMD patients of TREAT-NMD, Flanigan’s and GHCPAPF

Population	Mutation type	Reading-frame rule	Length of potential protein	Number of potential stop-gains	Joint prediction (Rules)	Joint prediction (Rules & patterns)
TREAT-NMD DMD patients	large deletions/duplications	5161/5681 = 90.8% NA 520/5681 = 9.2%	5283/5681 = 93.0% NA 398/5681 = 7.0%	5180/5681 = 91.2% NA 501/5681 = 8.8%	5419/5681 = 95.4% NA 262/5681 = 4.6%	5463/5681 = 96.2% NA 218/5681 = 3.8%
	small deletions/duplications	475/483 = 98.3% NA 8/483 = 1.7%	408/483 = 84.5% NA 75/483 = 15.5%	476/483 = 98.6% NA 7/483 = 1.4%	476/483 = 98.6% NA 7/483 = 1.4%	476/483 = 98.6% NA 7/483 = 1.4%
	splice sites	155/198 = 78.3% NA 43/198 = 21.7%	99/198 = 50.0% NA 99/198 = 50.0%	155/198 = 78.3% NA 43/198 = 21.7%	155/198 = 78.3% NA 43/198 = 21.7%	155/198 = 78.3% NA 43/198 = 21.7%
	total	5791/6362 = 91.0% NA 571/6362 = 9.0%	5790/6362 = 91.0% NA 572/6362 = 9.0%	5811/6362 = 91.3% NA 551/6362 = 8.7%	6050/6362 = 95.1% NA 312/6362 = 4.9%	6094/6362 = 96.8% NA 268/6362 = 4.2%
	nonsense		598/726 = 82.4% NA 128/726 = 17.6%	725/726 = 99.9% NA 1/726 = 0.1%	725/726 = 99.9% NA 1/726 = 0.1%	725/726 = 99.9% NA 1/726 = 0.1%
Flanigan’s DMD patients	large deletions/duplications	369/435 = 84.8% 23/435 = 5.3% 43/435 = 9.8%	374/435 = 86.0% 31/435 = 7.1% 30/435 = 6.9%	370/435 = 85.1% 24/435 = 5.5% 41/435 = 9.4%	386/435 = 88.7% 31/435 = 7.1% 18/435 = 4.1%	389/435 = 89.4% 32/435 = 7.4% 14/435 = 3.2%
	small deletions/duplications	69/78 = 88.5% 7/78 = 9.0% 2/78 = 2.6%	59/78 = 75.6% 4/78 = 5.1% 15/78 = 19.2%	69/78 = 88.5% 7/78 = 9.0% 2/78 = 2.6%	69/78 = 88.5% 7/78 = 9.0% 2/78 = 2.6%	69/78 = 88.5% 7/78 = 9.0% 2/78 = 2.6%
	splice sites	17/22 = 77.3% 1/22 = 4.5% 4/22 = 18.2%	12/22 = 54.5% 1/22 = 4.5% 9/22 = 40.9%	17/22 = 77.3% 1/22 = 4.5% 4/22 = 18.2%	17/22 = 77.3% 1/22 = 4.5% 4/22 = 18.2%	17/22 = 77.3% 1/22 = 4.5% 4/22 = 18.2%
	total	455/535 = 85.0% 31/535 = 5.8% 49/533 = 9.2%	445/535 = 83.2% 36/535 = 6.7% 54/535 = 10.1%	456/535 = 85.2% 32/535 = 6.0% 47/535 = 8.8%	472/535 = 88.2% 39/535 = 7.3% 24/535 = 4.5%	475/535 = 88.8% 40/535 = 7.5% 20/535 = 3.7%
	nonsense		146/206 = 70.9% 31/206 = 15.0% 29/206 = 14.1%	176/206 = 85.4% 0/206 = 0% 30/206 = 14.6%	176/206 = 85.4% 0/206 = 0% 30/206 = 14.6%	176/206 = 85.4% 0/206 = 0% 30/206 = 14.6%
GHCPAPF DMD patients	large deletions/duplications	213/231 = 92.2% 0/231 = 0% 18/231 = 7.8%	217/231 = 93.9% 5/231 = 2.2% 9/231 = 3.9%	217/231 = 93.9% 0/231 = 0% 14/231 = 6.1%	218/231 = 94.4% 5/231 = 2.2% 8/231 = 3.5%	218/231 = 94.4% 6/231 = 2.6% 7/231 = 3.0%
	small deletions/duplications	15/17 = 88.2% 1/17 = 5.9% 1/17 = 5.9%	13/17 = 76.5% 1/17 = 5.9% 3/17 = 17.6%	15/17 = 88.2% 1/17 = 5.9% 1/17 = 5.9%	15/17 = 88.2% 1/17 = 5.9% 1/17 = 5.9%	15/17 = 88.2% 1/17 = 5.9% 1/17 = 5.9%
	splice sites	1/1 = 100.0% 0/1 = 0% 0/1 = 0%	1/1 = 100.0% 0/1 = 0% 0/1 = 0%	1/1 = 100.0% 0/1 = 0% 0/1 = 0%	1/1 = 100.0% 0/1 = 0% 0/1 = 0%	1/1 = 100.0% 0/1 = 0% 0/1 = 0%
	total	229/249 = 92.0% 1/249 = 0.4% 19/249 = 7.6%	231/249 = 92.8% 6/249 = 2.4% 12/249 = 4.8%	233/249 = 93.6% 1/249 = 0.4% 15/249 = 6.0%	234/249 = 94.0% 6/249 = 2.4% 9/249 = 3.6%	234/249 = 94.0% 7/249 = 2.8% 8/249 = 3.2%
	nonsense		18/19 = 94.7% 1/19 = 5.3% 0/19 = 0%	19/19 = 100.0% 0/19 = 0% 0/19 = 0%	19/19 = 100.0% 0/19 = 0% 0/19 = 0%	19/19 = 100.0% 0/19 = 0% 0/19 = 0%

This table shows the statistics for large deletions/duplications, small deletions/duplications, splice sites, as well as nonsense mutations for TREAT-NMD, Flanigan’s and GHCPAPF data. FPR, false positive rate. FNR, false negative rate. Joint prediction (Rules): the joint prediction used the three rules, i.e., reading-frame rule, length of potential protein, number of potential stop-gains, and If any one is a positive judgment, the result is positive. Joint prediction (Rules & patterns): the joint prediction used the three rules and supplemental patterns on location of mutations

The joint prediction uses all the above three methods. Two criteria were used to determine the joint judgment. First, if a positive judgment comes from two of the three methods, the result is regarded as positive (i.e., DMD/DMD/BMD will be judged as DMD). By this criterion, the accuracy, FPR and FNR were 93.9%, NA, 6.1% (large deletions/duplications in 5341/5681 = 94.0% for accuracy, NA for FPR, 340/5681 = 6.0% for FNR; small deletions/duplications in 476/483 = 98.6% for accuracy, NA for FPR, 7/483 = 1.4% for FNR; splice sites in 155/198 = 78.3% for accuracy, NA for FPR, 43/198 = 21.7% for FNR) in TREAT-NMD DMD patients. They were 85.0, 6.0, 9.0% (large deletions/duplications in 369/435 = 84.8% for accuracy, 24/435 = 5.5% for FPR, 42/435 = 9.7% for FNR; small deletions/duplications in 69/78 = 88.5% for accuracy, 7/78 = 9.0% for FPR, 2/78 = 2.6% for FNR; splice sites in 17/22 = 77.3% for accuracy, 1/22 = 4.5% for FPR, 4/22 = 18.2% for FNR) in Flanigan’s DMD patients. In the GHCPAPF group they were 93.6, 0.4, and 6.0% (large deletions/duplications in 217/231 = 93.9% for accuracy, 0/231 = 0% for FPR, 14/231 = 6.1% for FNR; small deletions/duplications in 15/17 = 88.2% for accuracy, 1/17 = 5.9% for FPR, 1/17 = 5.9% for FNR; splice sites in 1/1 = 100.0% for accuracy, 0/1 = 0% for FPR, 0/1 = 0% for FNR). Second, if we get a positive judgment based on one of the three methods, the result is regarded as positive. The accuracy, FPR and FNR would increase to 95.1%, NA, and 4.9% (large deletions/duplications in 5419/5681 = 95.4% for accuracy, NA for FPR, 262/5681 = 4.6% for FNR; small deletions/duplications in 476/483 = 98.6% for accuracy, NA for FPR, 7/483 = 1.4% for FNR; splice sites in 155/198 = 78.3% for accuracy, NA for FPR, 43/198 = 21.7% for FNR) in TREAT-NMD DMD patients. They were 88.2, 7.3, and 4.5% (large deletions/duplications in 386/435 = 88.7% for accuracy, 31/435 = 7.1% for FPR, 18/435 = 4.1% for FNR; small deletions/duplications in 69/78 = 88.5% for accuracy, 7/78 = 9.0% for FPR, 2/78 = 2.6% for FNR; splice sites in 17/22 = 77.3% for accuracy, 1/22 = 4.5% for FPR, 4/22 = 18.2% for FNR) in Flanigan’s DMD patients. In GHCPAPF group they were 94.0, 2.4, 3.6% (large deletions/duplications in 218/231 = 94.4% for accuracy, 5/231 = 2.2% for FPR, 8/231 = 3.5% for FNR; small deletions/duplications in 15/17 = 88.2% for accuracy, 1/17 = 5.9% for FPR, 1/17 = 5.9% for FNR; splice sites in 1/1 = 100.0% for accuracy, 0/1 = 0% for FPR, 0/1 = 0% for FNR). We took this result as the “joint prediction (Rules)” in Table 2.

For nonsense mutations, the accuracy of protein length, stop-gain number and the joint prediction for TREAT-NMD DMD patients was 82.4, 99.9 and 99.9%, respectively; for Flanigan’s DMD patients they were 70.9, 85.4 and 85.4%, respectively. The accuracy for DMD patients of GHCPAPF was 94.7, 100.0 and 100.0% respectively. The FPR and FNR of protein length, stop-gain number and the joint prediction were shown in Table 2. After application of ESE rule [10, 11] the accuracy of ESE disrupted mutations among DMD patients in TREAT-NMD, Flanigan and GHCPAPF dropped to 0/70 = 0%, 6/49 = 12.2% and 0/5 = 0%, respectively. Therefore, the ESE rule was not used in the DMDtoolkit.

For large deletions, several supplemental patterns to the reading-frame rule were applied. It was reported that in-frame deletions within exons 2–8 caused severe BMD, whereas deletions in the major hotspot generally caused typical BMD [21, 22]. In-frame deletions removing both the actin-binding domain and part of the central rod domain usually cause DMD [8, 20, 23]. Large in-frame deletions in the central rod domain removing more than 35 exons usually led to DMD [8], while deletions of no more than 35 exons likely led to BMD [8, 24, 25]. Mutations in the cysteine-rich domain usually resulted in DMD [17, 18] whereas deletions in the syntrophin-binding domain (exons 71-74) were reported in some BMD patients and mutations located in exon 74 or behind it were found in both BMD and DMD patients [17, 26]. Compared to the joint prediction without conducting the supplementary patterns, the accuracy, FPR, FNR with application of patterns was 100% (16/16), NA, 0% (0/16) in DMD patients of TREAT-NMD; was 80.0% (4/5), 20.0% (1/5), 0% (0/5) in DMD patients of Flanigan. They were 50.0% (1/2), 50.0% (1/2), 0% (0/2) in DMD patients of GHCPAPF (see sheet “Supplementary patterns” in Additional file 3 “prediction results of DMD patients.xlsx” for details).

The accuracy, FPR, FNR of the six and seven combinations of multiple mutations in Flanigan and GHCPAPF were 83.3% (5/6), 0% (0/6), 16.7% (1/6), and 85.7% (6/7), 0% (0/7), 14.3% (1/7), respectively. While the accuracy, FPR, FNR of reading-frame rule was 83.3% (5/6), 0% (0/6), 16.7% (1/6) and 42.9% (3/7), 0% (0/7), 57.1% (4/7) in Flanigan and GHCPAPF, respectively. Please see the sheet “Multiple mutations” in Additional file 3 “prediction results of DMD patients.xlsx” for details.

### Visualization

**Fig. 2:**
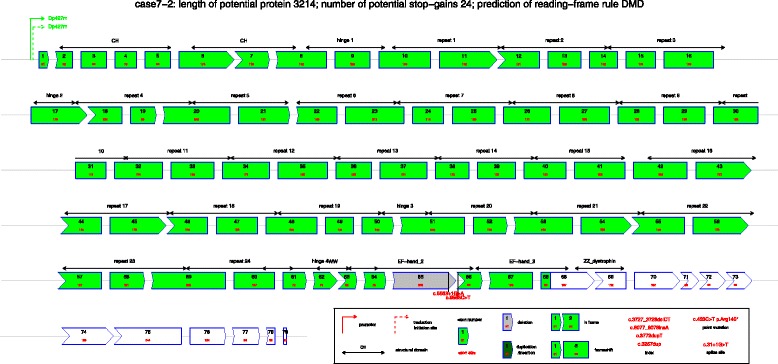
An example of combination of mutations. The mutations include a splice site mutation nearby exon 65 (c.9563+1G>A) and a nonsense mutation in exon 66 (c.9568C>T), both of which are common mutation types in TREAT-NMD database. However, the exon-skipping of exon 65 will change the nonsense mutation to a missense mutation

**Fig. 3:**
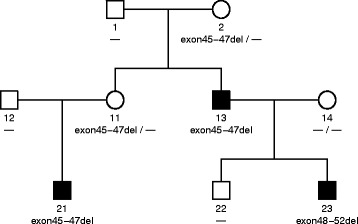
A pedigree of BMD father and DMD son. The family has inherited a disease-causing mutation, i.e., Exon45-47del, which led to BMD. Individual 13 was the proband, the BMD father. Individual 23 was his DMD son with a novo mutation of exon48-52del

**Fig. 4:**
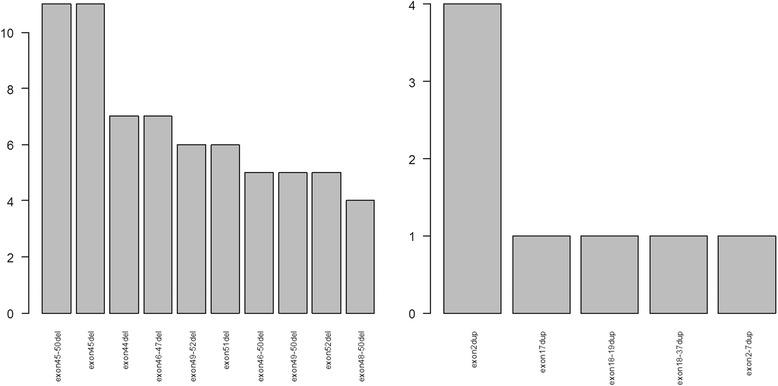
Histogram of top 10 exon deletions and top 5 exon duplications. The two graphs were plotted by the commands plot.freq(“del”,10) and plot.freq(“dup”,5) respective in a R console

DMDtoolkit can draw the sequence of a mutant protein and turn the document into a pdf file (Fig. 2). For protein with multiple mutations resulting in more than two frameshifts, it is difficult to apply the reading-frame rule to predict the mutant protein because the stop-gain may happen before the second frameshift, or the upstream frameshift may change the downstream missense to nonsense. Visualization is an easy way to show the change of a mutated protein, such as c.9563+1G>A plus c.9568C>T (Fig. 2). The Additional file 3 “results_DMDtoolkit/results_diagnosis/*.pdf”, such as “case7-1 (combination of multiple mutations).pdf”, showed examples of the seven mutation types. DMDtoolkit expanded the R package “kinship” [27] to draw multiple pedigrees at once (Fig. 3). DMDtoolkit can also draw the top N mutations’ distribution (N can be set via command parameter) (Fig. 4).

## Discussion

According to the first criterion, the joint prediction method was 1.9, 0 and 1.6% higher than the reading-frame rule on accuracy of DMD patients from TREAT-NMD, Flanigan’s and GHCPAPF groups respectively. According to the second criterion, the joint prediction was 4.1, 3.2 and 2.0% more than the reading-frame rule on accuracy of DMD patients from TREAT-NMD, Flanigan’s and GHCPAPF, respectively. The improvement of accuracy mainly originated from the decrease of FNR in DMD patients with large deletions/duplications, and it benefited from the length of potential protein method.

For large deletions, the application of the supplemental patterns improved the total accuracy of joint prediction method without patterns (i.e., “joint prediction (Rules)” in Table 2) up by 1.7, 0.3, 0% and up to 96.8, 88.8 and 94.0% in DMD patients of TREAT-NMD, Flanigan and GHCPAPF, respectively. The improvement was due to the identification of in-frame deletions removing both the actin-binding domain and part of the central rod domain which usually cause DMD.

Future plans for development include the integration of data on pathways and protein-protein interaction (PPI) networks [28]. These will allow more comprehensive analyses on the biological processes of dystrophin and its interactive genes. An automated machine learning approach [29] will also be exploited to quantitatively predict the procession of disease using all available risk/benefit indicators as well as the probability of BMD/IMD/DMD.

## Conclusions

DMDtoolkit is a unique computer software specifically developed to provide an easy way to analyze the mutant dystrophin protein in order to predict the diagnosis of DMD/BMD. This is achieved by combining genomic analysis with a bioinformatic approach. As for the prediction of DMD/BMD, DMDtoolkit provides a unique advantage when compared with previous predictions solely based on the reading-frame rule. It can automatically and rapidly predict clinical phenotypes even in the presence of multiple mutations. The accuracy of the current joint method is about 3% more than that of reading-frame rule alone. Its advantage is due to the bioinformatics approach combining the three different methods for prediction.

Basic statistics include calculation of summary, correlation coefficient, regression coefficient, and *t* test (in the Additional file 3 “supplement1.docx” in the folder “results_DMDtoolkit/results_statistics & graph/”). Basic graphs include pedigree, histogram, scatter plot with trend line, stem and leaf plot, and cluster dendrogram (in the Additional file 3 “supplement2.docx” in the folder “results_DMDtoolkit/results_statistics & graph/”). These results can help patients and clinicians more easily understand the disease and detect risk/benefit indicators.

## Availability of data and materials

**Project name**: DMDtoolkit.

**Project home page**: https://github.com/zhoujp111/DMDtoolkit or http://www.dmd-registry.com/xzzq/index.jhtml.

**Archived version**: 1.0.

**Operating system(s)**: Platform independent.

**Programming language**: R and Perl.

**Other requirements**: R 3.0 or higher, ActivePerl 5.16 or higher.

**License**: GNU GPL.

**Any restrictions to use by non-academics**: licence needed.

## Additional files

Additional file 1: codes_DMDtoolkit. This folder includes all the codes, manual (“Manual.docx”) and required databases/annotations of DMDtoolkit. For example, “ESE matrices.txt” contains the matrices of serine/arginine-rich (SR) proteins. (RAR 2602 kb)
Additional file 2: data_DMDtoolkit. This folder includes all the data used in the present manuscript, such as “Flanigan’s_DMD_patients.txt” for aided diagnosis for DMD/BMD, “DMDsamples.txt” for drawing mutated protein, and “pedigree.txt” for drawing pedigree of DMD family. (RAR 28 kb)
Additional file 3: results_DMDtoolkit. This folder includes all the results produced by DMDtoolkit, such as “prediction results of DMD patients.xlsx” which contains the original prediction results of DMD patients from TREAT-NMD, Flanigan’s and GHCPAPF, “case7-1 (combination of multiple mutations).pdf” which is a vector illustration of a combination of multiple mutations. “supplement1.docx”. Examples of basic statistics includes calculation of summary, correlation coefficient, regression coefficient, and *t* test. “supplement2.docx”. Examples of basic graphs include pedigree, histogram, scatter plot with trend line, stem and leaf plot, and cluster dendrogram. (RAR 5435 kb)

## Abbreviations

BMD: Becker muscular dystrophy
BMI: Body mass index
DMD: Duchenne muscular dystrophy
ESE: Exonic splicing enhancer
FNR: False negative rate
FPR: False positive rate
GHCPAPF: General Hospital of Chinese People’s Armed Police Forces
GNU: GNU’s Not Unix
IMD: Intermediate muscular dystrophy
LVEDD: Left ventricular end-diastolic dimension
Perl: Practical extraction and reporting language
PPI: Protein-protein interaction
SNIP: Sniff nasal inspiratory pressure
SR: Serine/arginine-rich
WISC: Wechsler Intelligence Scale for Children
